# Optical recording reveals topological distribution of functionally classified colorectal afferent neurons in intact lumbosacral DRG

**DOI:** 10.14814/phy2.14097

**Published:** 2019-05-13

**Authors:** Tiantian Guo, Zichao Bian, Kyle Trocki, Longtu Chen, Guoan Zheng, Bin Feng

**Affiliations:** ^1^ Department of Biomedical Engineering University of Connecticut Storrs Connecticut

**Keywords:** Calcium imaging, colon, electrophysiology, GCAMP, single‐unit

## Abstract

Neuromodulation as a non‐drug alternative for managing visceral pain in irritable bowel syndrome (IBS) may target sensitized afferents of distal colon and rectum (colorectum), especially their somata in the dorsal root ganglion (DRG). Developing selective DRG stimulation to manage visceral pain requires knowledge of the topological distribution of colorectal afferent somata which are sparsely distributed in the DRG. Here, we implemented GCaMP6f to conduct high‐throughput optical recordings of colorectal afferent activities in lumbosacral DRG, that is, optical electrophysiology. Using a mouse ex vivo preparation with distal colorectum and L5‐S1 DRG in continuity, we recorded 791 colorectal afferents' responses to graded colorectal distension (15, 30, 40, and 60 mmHg) and/or luminal shear flow (20–30 mL/min), then functionally classified them into four mechanosensitive classes, and determined the topological distribution of their somata in the DRG. Of the 791 colorectal afferents, 90.8% were in the L6 DRG, 8.3% in the S1 DRG, and only 0.9% in the L5 DRG. L6 afferents had all four classes: 29% mucosal, 18.4% muscular‐mucosal, 34% low‐threshold (LT) muscular, and 18.2% high‐threshold (HT) muscular afferents. S1 afferents only had three classes: 19.7% mucosal, 34.8% LT muscular, and 45.5% HT muscular afferents. All seven L5 afferents were HT muscular. In L6 DRG, somata of HT muscular afferents were clustered in the caudal region whereas somata of the other classes did not cluster in specific regions. Outcomes of this study can directly inform the design and improvement of next‐generation neuromodulation devices that target the DRG to alleviate visceral pain in IBS patients.

## Introduction

Patients with irritable bowel syndrome (IBS) typically exhibit reduced response thresholds to rectal distension, pain during normal bowel function, and increased tenderness and size in areas of somatic referral (i.e., hypersensitivity) (Feng et al. 2012a; Chen et al. 2017). The underlying mechanism(s) of this hypersensitivity is widely attributed to the impaired function of the brain–gut axis, particularly afferent sensitization, which appears necessary for the persistence of IBS‐related pain and hypersensitivity (Verne et al. 2003, 2005). Studies by us and others document the innervation of distal colorectum by four classes of mechanosensitive afferent fibers that are tuned to encode normal colorectal distension, mucosal shear stress, and/or high‐intensity mechanical probing (Brierley et al. 2004; Feng et al. 2010, 2012b,2012c; Feng and Gebhart 2011, 2015; La et al. 2012). Pharmacological approach for managing visceral pain by reversing peripheral afferent sensitization has limited success largely due to the off‐target side effects (Chen et al. 2017). In contrast, peripheral neuromodulation significantly limits the off‐target effects by focal delivery of physical energy to peripheral nerves (Chakravarthy et al. 2016), nerve roots (Foreman and Linderoth 2012; Zhang et al. 2014) and dorsal root ganglion (DRG) (Krames 2015; Liem et al. 2016). Thus, neuromodulation that targets sensitized colorectal afferents, especially their somata in the DRG has the potential as a non‐drug alternative for managing visceral pain.

Colorectal afferent somata in mouse DRG have been labeled by neural tracing studies revealing that mouse distal colorectum is innervated by T13‐L1 thoracolumbar and L5‐S1 lumbosacral DRG (Christianson et al. 2006b), of which the lumbosacral innervation appears necessary for encoding and transmitting visceral nociception (Kyloh et al. 2011). In lumbosacral innervation, mouse colorectal neurons are concentrated in the L6 DRG, and are sporadic in the adjacent L5 and S1 DRG (Christianson et al. 2006b). Even in L6 DRG, colorectal neurons are the minorities contributing to <10% total DRG neurons, and appear to sparsely spread throughout the DRG without clustering in specific regions (La et al. 2011, 2016). The vast majority of colorectal DRG neurons are small and medium in diameter, immunohistologically positive for TRPV1, CGRP, and NaV1.8 (Beyak et al. 2004; Christianson et al. 2006a; Malin et al. 2009; Beyak 2010). Unlike the cutaneous DRG neurons that can be distinctly divided into peptidergic (positive for calcitonin gene‐related peptide [CGRP]) and non‐peptidergic (positive for isolectin B4 [IB4]) populations (Stucky and Lewin 1999; Joseph and Levine 2010), a significant proportion of colorectal neurons are positive for both CGPR and IB4 (La et al. 2016). A recent mRNA sequencing study has further divided colorectal neurons into seven categories based upon their messenger RNA profiles (Hockley et al. 2018).

Despite the aforementioned body of literature regarding the anatomy, molecular profiles, and neural encoding functions of colorectal afferents, further advancement of DRG neuromodulation for managing visceral pain requires knowledge on the topological distributions of functionally classified colorectal neurons in the DRG, which has not been investigated by prior studies. For instance, knowing the spatial location of high‐threshold colorectal afferents (putative nociceptors) in specific lumbosacral DRG will likely inform the design of DRG stimulation to selectively target colorectal nociceptors while sparing other sensory afferents that may play important physiological roles for the pelvic floor organs. Determining the topological distribution of colorectal DRG neurons requires functional recordings from intact ganglion, which is challenged by the small proportion and sparse distribution of colorectal neurons in the DRG, and has been achieved by only a handful of studies via intracellular recordings using a sharp quartz glass electrode (Malin et al. 2011; Hibberd et al. 2016). However, this approach is technically challenging and cannot efficiently record a large number of neurons to reveal the topological distributions of the colorectal neurons. Alternatively, the GCaMP6f, one of the recent genetically encoded calcium indicators allows recording of intracellular Ca^2+^ transients that correlate with individual neural action potentials (Chen et al. 2013), allowing “functional” characterization of those neurons via imaging their fluorescent GCaMP6f signals (Chisholm et al. 2018).

In this study, we developed an optical recording approach to characterize colorectal afferent functions in a mouse ex vivo preparation with distal colorectum, pelvic nerve, L5‐S1 DRG, and dorsal roots in continuity. We conducted fast imaging recordings (100 frames per second) of intracellular GCaMP6f signals in intact DRG to resolve the fast calcium transients in individual DRG somata, allowing functional characterization of colorectal DRG neurons using this imaging method, that is, optical electrophysiology. By this high throughput approach, we determined the responses of 791 neurons to mechanical colorectal distension and/or mucosal shear stress, functionally divided them into four classes, and identified their topological distributions in the lumbosacral DRG. Portions of these data have been reported in the abstract form (Feng et al. 2018).

## Materials and Methods

All experiments were reviewed and approved by the University of Connecticut Institutional Animal Care and Use Committee.

### Transgenic mice

The Ai95 mice (C57BL/6 background) carrying heterozygous GCaMP6f gene (strain# 28865, The Jackson Laboratory, CT) and homozygous VGLUT2‐Cre mice (strain# 28863, Jackson Laboratory, CT) were crossbred. The Ai95 mice carried the gene “CAG‐GCaMP6f” in the Gt(ROSA)26Sor locus, which was preceded by a LoxP‐flanked STOP cassette to prevent its expression. By crossing Ai95 mice with VGLUT2‐Cre mice, the Cre‐expressing cell population has the STOP cassette trimmed, resulting in the expression of GCaMP6f in glutamatergic neurons expressing type 2 vesicular glutamate transporter (VGLUT2), which made up the vast majority of sensory neurons innervating distal colon and rectum (colorectum) (Brumovsky et al. 2011). Offspring of both sexes aged 8–14 weeks with both heterozygous GCaMP6f and VGLUT2‐Cre genes (i.e., VGLUT2/GCaMP6f) were used for optical electrophysiological recordings.

To characterize the efficiency of VGLUT2‐Cre mice in driving the expression in colorectal sensory neurons, we crossed the VGLUT2‐Cre mice with a tdTomato reporter line (Ai14, strain#7914, The Jackson Laboratory, CT). Offspring with both heterozygous tdTomato (tdT) and VGLUT2‐Cre genes, that is, VGLUT2/tdT mice were used for anatomical tracing studies.

### Anatomic tracing of colorectal DRG neurons

All surgeries were aseptic; anesthesia was initiated and maintained with 4% and 2% isoflurane inhalation, respectively. In VGLUT2/tdT mice, primary afferent neurons innervating the colorectum were identified in DRG tissue sections by the content of the fluorescent retrograde tracer fast blue (FB; #NC0182483, Fisher Scientific, East Greenwich, RI). FB tracer injection was performed 7–28 days prior to harvesting DRG. Mice were anesthetized with isoflurane, the distal colon was exposed by laparotomy, and the colon wall was injected with FB (1 mg/100 *μ*L in sterile saline; 10 *μ*L total distributed into 2–4 sites) by a syringe with a 33‐gauge needle (model 7643‐01, Hamilton Company, Arlington, MA). Any leakage from injection sites was removed using a cotton‐tipped applicator and the peritoneal cavity was rinsed with sterile saline before suturing muscle and skin separately. To alleviate pain and distress, meloxicam (2 mg/kg, Boehringer Ingelheim Vetmedica, Duluth, Georgia) was given three times in 72 h for postoperative analgesia.

### Immunohistochemistry

As previously described (Feng et al. 2012c), VGLUT2/tdT mice were euthanized via CO_2_ inhalation. The lumbosacral (L6‐S1) DRG were harvested and fixed with 4% paraformaldehyde in 0.16 mol/L phosphate buffer containing 14% picric acid (Sigma‐Aldrich). After cryoprotection in 20% sucrose, fixed tissue was embedded in OCT compound (Sakura Finetek, Tokyo, Japan), frozen, and sectioned at 10 *μ*m. Tissue sections were incubated with a rabbit antibody against the red fluorescent protein (1:1000, Rockland, Limerick, PA) and the signals were further amplified by Alexa Fluor^®^ 594‐conjugated anti‐rabbit IgG (1:200, Abcam, Cambridge, MA).

Fast Blue‐containing and immunostained DRG neurons were identified and quantified as detailed previously (La et al. 2016). Briefly, the 8‐bit grayscale immune‐images were set with a threshold that excluded at least 99.99% of background intensity based on its Gaussian distribution using ImageJ (version 1.8.0; NIH). The threshold value was calculated from the standard normal distribution (*Z*) equation: threshold = 3.72 SD + m, where 3.72 was the *Z*‐score at *P* = 0.9999, SD and m were the Standard Deviation and mean of background intensities in 8‐bit grayscale (0–255), respectively.

### Ex vivo preparation of colorectum‐pelvic nerve‐DRG

Mice of the correct genotype 8–14 weeks of age were deeply anesthetized by intraperitoneal and intramuscular injection of a 0.4 mL cocktail of ketamine (120 mg/kg) and xylazine (10 mg/kg). Mice were then euthanized by perfusion from the left ventricle with modified ice‐cold Krebs solution replacing sodium chloride with equal molar of sucrose (in mmol/L: 236 Sucrose, 4.7 KCl, 25 NaHCO_3_, 1.3 NaH_2_PO_4_, 1.2 MgSO_4∙_7H_2_O, 2.5 CaCl_2_, 11.1 d‐Glucose, 2 butyrate, 20 acetate, 0.004 nifedipine and 0.003 indomethacin) bubbled with carbogen (95% O_2_, 5% CO_2_). A dorsal laminectomy was performed to expose the thoracic and lumbar spinal cord. The colorectum with attached S1, L6, and L5 spinal nerve and DRG was carefully dissected via blunt dissection, and transferred to a tissue chamber superfused with 32–34°C Krebs solution (in mmol/L: 117.9 NaCl, 4.7 KCl, 25 NaHCO_3_, 1.3 NaH_2_PO_4_, 1.2 MgSO_4∙_7H_2_O, 2.5 CaCl_2_, 11.1 d‐Glucose, 2 butyrate, 20 acetate, 0.004 nifedipine and 0.003 indomethacin) bubbled with carbogen (95% O_2_, 5% CO_2_), consistent with our prior ex vivo studies on colorectal afferents (Feng and Gebhart 2011; Feng et al. 2016). The colorectum was cannulated and connected to a custom‐built distending device consisting of computer‐controlled fluid valves and hydrostatic pressure columns of 15, 30, 45, and 60 mmHg filled with phosphate buffered saline (PBS).

### Optical recording of evoked fluorescent GCaMP6f signal

We employed an upright microscope platform (BX51WI, Olympus, Waltham, MA) to capture one whole DRG with a water immersion 10× objective (UMPLFLN 10XW, 0.3 NA) using a regular halogen epi‐illumination light source and a regular filter cube for sample illumination. A high‐speed ultra‐low noise sCMOS camera (Xyla‐4.2P, 82% quantum efficiency, Andor Technology, South Windsor, CT) was used to capture high‐resolution images (1920 × 1920, 2 × 2 bin) at 100 frames per second, which provided a spatial resolution of 1.6 pixels/*μ*m sufficient to resolve individual DRG neurons. This system allowed the recording of Ca^2+^ transients to resolve individual action potentials (APs) in multiple GCaMP6f‐expressing DRG neurons simultaneously.

We implemented an image analysis algorithm to automatically locate activated DRG neurons that had evoked APs (i.e., Ca^2+^ transients) based on intensity fluctuation across different frames. We performed marker‐based watershed segmentation to label different cells (Yang et al. 2006), allowing automated recording of GCaMP6f Ca^2+^ signals in multiple DRG neurons. We integrated the image analysis algorithm into a plug‐in program to work with Micro‐Manager, an open‐source microscopy software sponsored by the NIH (Edelstein et al. 2014). Using this image recording approach, colorectal DRG neurons were functionally characterized in intact L5‐S1 DRG at high efficiency.

### Validation of GCaMP transients as individual action potentials

To validate that image recordings of GCaMP transients correlated with individual APs, we conducted concurrent recordings of GCaMP signals and intracellular electrical potentials in intact DRG attached with dorsal roots. Action potentials were evoked by electrical stimulation of the dorsal root with a suction electrode (1 mA monopolar cathodic, 0.1 msec duration @ 0.5–4 Hz). In addition to the optical recording of GCaMP6f signal, DRG neurons with positive GCaMP6f response were individually impaled with a high impedance (100–300 MΩ) quartz electrode filled with 1.0 mol/L potassium acetate for intracellular recordings of the cell potential by a patch‐clamp amplifier (Multiclamp 700A, Molecular Devices, San Jose, CA). The APs were digitized at 20 kHz and stored by a Power 1401 interface (Cambridge Electronic Design), which was also programmed to trigger the GCaMP6f image recordings to ensure proper alignment of action potentials with GCaMP6f imaging data.

### Afferent identification and classification

Mouse colorectal afferents were activated by two physiologically‐correlated stimuli at the colorectum: stepped luminal distension by hydrostatic fluid column of phosphate buffered saline (PBS, 15, 30, 45, and 60 mmHg of 5‐sec steps) and luminal shear flow of PBS (20–30 mL/min). Based upon response profiles to graded distension and luminal shear, colorectal DRG were functionally classified into four classes: low‐threshold (LT) muscular, high‐threshold (HT) muscular, mucosal, and muscular‐mucosal classes. LT‐muscular afferents responded to all four distension pressure levels whereas HT‐muscular only responded to noxious distension pressure (30, 45, and 60 mmHg); colorectal intraluminal pressure beyond 20 mmHg is considered noxious to mice (Kamp et al. 2003; Feng et al. 2010). Mucosal afferents did not respond to distension but responded to luminal shear flow. Muscular‐mucosal afferents responded to both luminal shear flow and colorectal distension at all four pressure levels.

### Data recording and analysis

Extracted GCaMP6f signals in the form of pixel intensity (0–255) from individual DRG neurons were normalized by the pre‐stimulus intensity. Peak GCaMP6f transients were determined when the signal increased by 3% within 200 msec. Proportions of afferent classes were compared by Chi‐square test using SigmaPlot v11.0 (Systat software, Inc., San Jose, CA). A value of *P* < 0.05 was considered significant.

## Results

**Figure 1:**
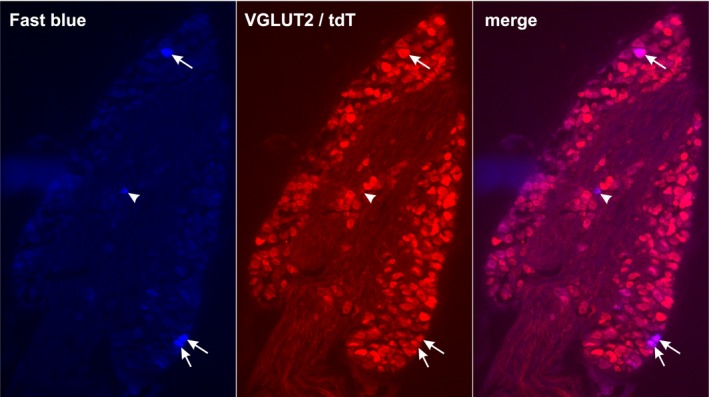
VGLUT2‐Cre efficiently drives gene expression in colorectal DRG neurons. Neural tracer Fast Blue (FB) injected into the colon wall was retrogradely transported to the lumbosacral DRG as shown in the blue channel. The tdTomato (tdT) expression driven by VGLUT2‐Cre was stained by an RFP antibody as shown in the red channel. White arrows indicate colorectal DRG neurons (FB‐positive) that are immuno‐positive to tdTomato, and white arrowheads indicate colorectal DRG neurons immuno‐negative to tdTomato.

In three male and three female VGLUT2/tdTomato mice, the colorectal neurons in the lumbosacral DRG (L5 to S1) were labeled by the neural tracer fast blue (FB) as shown in Figure 1. Among the 87 FB‐positive colorectal neurons (white arrows or arrowheads in Fig. 1), 78% (68/87) showed positive immunoreactivity to tdTomato as indicated by the white arrows in Figure 1. The portions of tdTomato‐positive neurons from male (36/47) and female mice (32/40) were comparable and thus neurons from both sexes were pooled together. This confirms the previous finding that the majority of colorectal DRG neurons express VGLUT2 and the VGLUT2‐Cre efficiently drives the gene expression in lumbosacral colorectal afferents (Brumovsky et al. 2011). Immunostaining results also show that only 68 of the 2060 (3.3%) tdTomato‐positive DRG neurons from 15 DRG (L5 to S1) are FB‐positive colorectal neurons, which confirms that the colorectal neurons are sparsely distributed in lumbosacral DRG.

**Figure 2:**
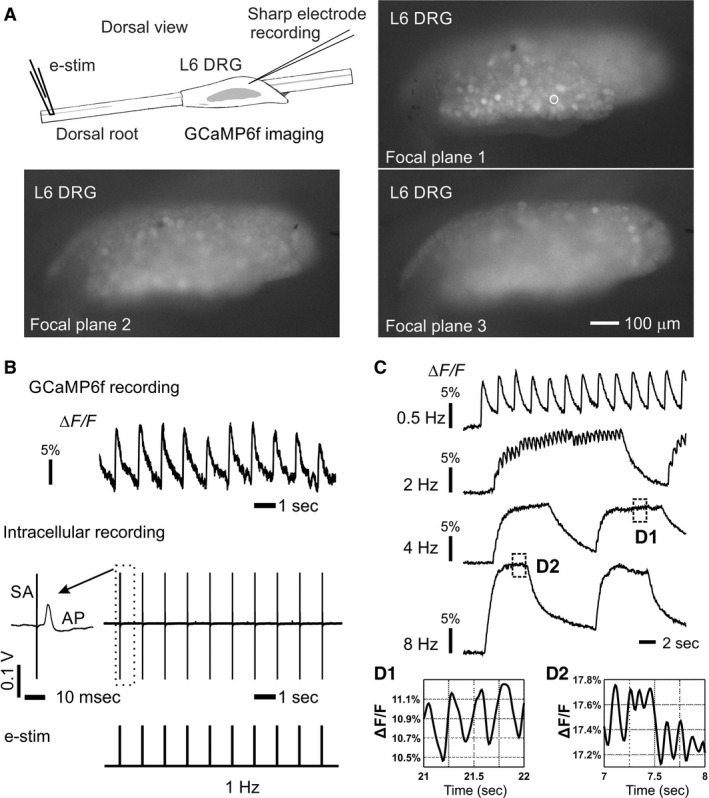
Concurrent GCaMP6f image recording and intracellular sharp electrode recording at L6 DRG. (A) Electrical stimulation was applied to the attached dorsal root via a suction electrode (0.1 msec duration, 1–3 mA) and evoked action potentials were recorded at the DRG both optically by a fluorescent microscope (10 × water immersion objective, 0.3 NA) and electrophysiologically by a penetrating sharp quartz glass electrode (100–300 MΩ impedance). A whole DRG can be recorded with a spatial resolution of the GCaMP6f signals at individual DRG neurons (white circle). Optical recordings were conducted at multiple focal planes 50 *μ*m apart to account for the contoured DRG surface. (B)The close correlation of GCaMP6f transients with individual action potentials at 1 Hz stimulation. (C) GCaMP6f signals evoked by electrical stimulations of different frequencies (0.5–8 Hz). (D) Expanded view of the GCaMP6f signals at 4 and 8 Hz stimulation. The GCaMP6f fluorescent signal was normalized by the average intensity and denoted as *ΔF/F*.

As illustrated in Figure 2A, GCaMP6f image recording and intracellular sharp electrode recording were conducted concurrently to assess whether GCaMP6f signals correlate with neural activities. Optical recordings were conducted at multiple focal planes 50 *μ*m apart to ensure the capture of GCaMP6f signals throughout the contour of the DRG in the dorsal lateral view. As shown in Figure 2B, the GCaMP6f transients closely correlate with individual action potentials evoked by 1 Hz electrical stimulation at the dorsal root. Stimulation was assessed at higher frequencies as shown in Figure 2C, and the GCaMP6f signal is capable to resolve individual action potential spikes at up to 4 Hz as shown in Figure 2D1. As shown in Figure 2D2, the individual spikes cannot be reliably detected at a stimulus frequency of 8 Hz, but the elevated magnitude of the fluorescent signal provides a qualitative estimation of the afferent firing frequency (compare the 4 and 8 Hz signals in Fig. 2C).

**Figure 3:**
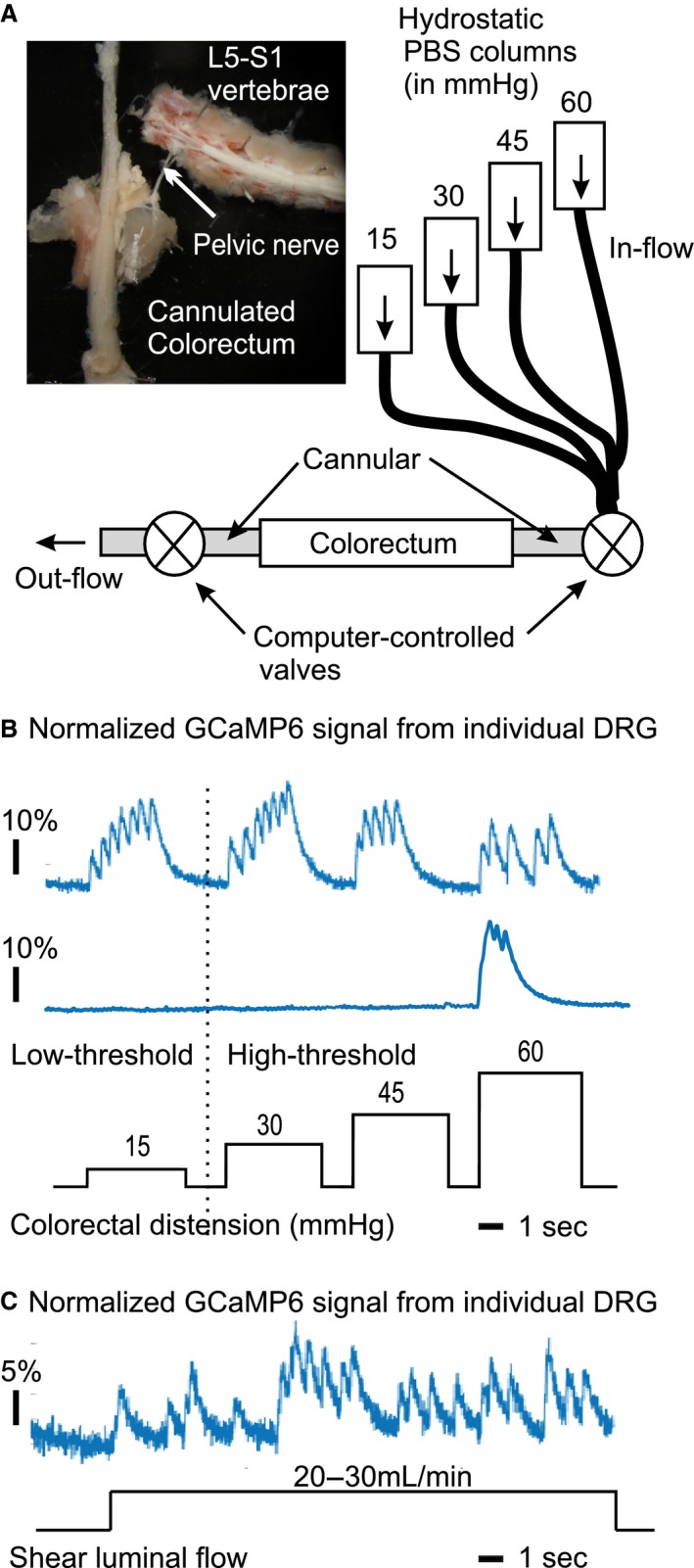
Functional characterization of colorectal afferents via GCaMP6f DRG recordings in an ex vivo colorectum‐pelvic nerve‐DRG preparation. (A) The distal colorectum, pelvic nerve and lumbosacral DRG (L5 to S1) were harvested in continuity. The colorectum was cannulated and subjected to two computer‐controlled mechanical stimuli: graded colorectal distension (15, 30, 45, 60 mmHg, each for 5 sec) and luminal shear flow (20–30 mL/min). Displayed in (B) and (C) are GCaMP6f recordings of afferent responses to graded colorectal distension and luminal shear flow, respectively.

Figure 3A illustrates an ex vivo colorectum‐pelvic nerve‐DRG preparation, in which afferent endings in the colorectum were subjected to two computer‐controlled mechanical stimuli: graded colorectal distension by hydrostatic pressure (15–60 mmHg) and luminal shear flow (20–30 mL/min). As shown in Figure 3B, colorectal afferents' responses to graded colorectal distension were captured by GCaMP6f signals from individual DRG neurons. Distending pressure beyond 20 mmHg was considered noxious (Feng et al. 2010), and afferents with response threshold below and above 20 mmHg were categorized as low‐threshold (LT) and high‐threshold (HT), respectively. Displayed in Figure 3C is a typical colorectal afferent response to luminal shearing flow, which is equivalent to fine stroking of the mucosal surface in prior studies (Feng and Gebhart 2011, 2015).

**Figure 4:**
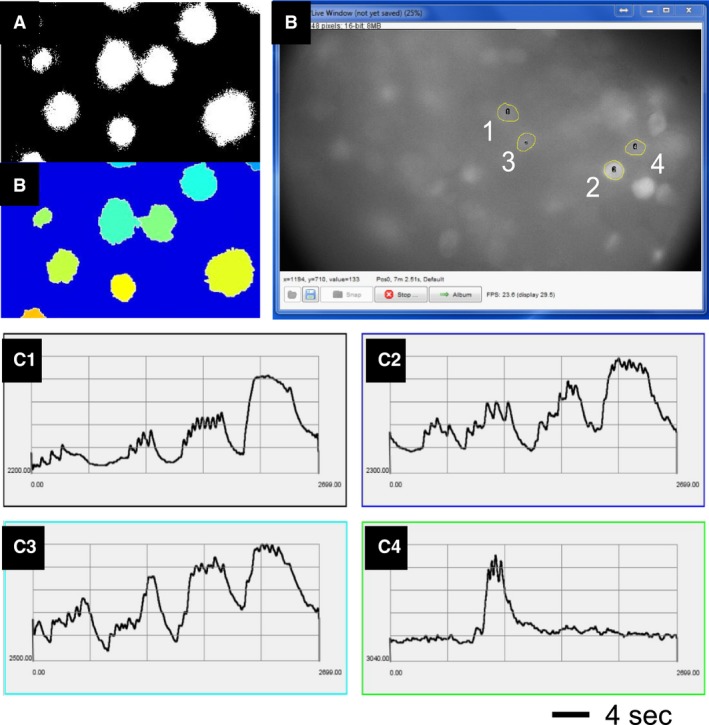
Automatic extraction of GCaMP6f transients from image sequences each containing 2700 images by the custom‐built plug‐in software for Micro‐Manager. (A) The boundary of the neurons was automatically determined by marker‐based watershed segmentation. (B) Neurons with intensity fluctuation beyond the detection threshold were automatically labeled. (C) GCaMP6f transients extracted from the four labeled neurons in (B).

For either colorectal distension or luminal shearing flow tests, GCaMP6f images were continuously recorded for 27 sec at 100 frames/sec, generating a sequence of 2700 images. Figure 4 illustrates the custom‐built plug‐in software to Micro‐Manager (Edelstein et al. 2014) that automatically extracts GCaMP6f transients from activated DRG neurons in those image sequences. After automatic detection of the neural boundaries by marker‐based watershed segmentation in Figure 4A, activated neurons with intensity fluctuation beyond the detection threshold were automatically labeled for extraction of GCaMP6f transients in Figure 4B. Figure 4C shows the fluorescence intensity signals from the labeled regions in Figure 4B.

**Figure 5:**
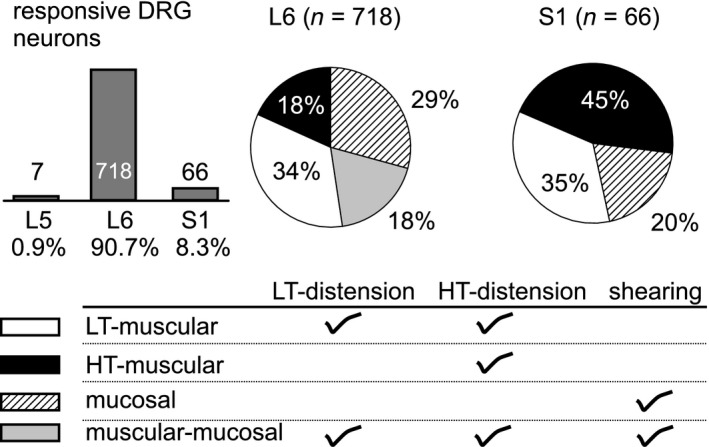
Functional classification of 791 lumbosacral colorectal afferents from 8 male and 6 female VGLUT2/GCaMP6f mice. Afferents responding to mechanical colorectal distension or shear flow were predominantly in L6 DRG (90.7%), and also found in S1 (8.3%) and L5 DRG (0.9%). All four afferent classes were present in L6 DRG whereas muscular‐mucosal afferents were absent in S1 DRG. LT, low threshold; HT, high threshold. Data from both sexes were pooled together.

As shown in Figure 5, a total of 791 lumbosacral DRG neurons from eight male and six female VGLUT2/GCaMP6f mice presented evoked GCaMP6f responses to either colorectal distension or luminal shear flow, of which the vast majority (90.7%) were in L6 DRG and the rest in S1 DRG. Only 8.3% colorectal afferent neurons were in the S1 DRG and almost no neurons were recorded in L5 DRG (0.9%, 7/791). Based upon the response profiles to low‐ or high‐threshold (LT‐ or HT‐) distension and luminal shearing, the colorectal afferents were divided into four classes: LT‐muscular, HT‐muscular, mucosal and muscular‐mucosal. All four afferent groups were present in the L6 DRG whereas muscular‐mucosal afferents were absent in the S1 DRG. The seven L5 afferents were all HT‐muscular. Among the 791 neurons, 472 were from male mice and 319 from female mice. There was no difference between males and females in either the DRG segment distributions (92% vs. 91% in L6, 8% vs. 9% in S1, *P* = 0.60) or the proportions of the four afferent classes (28% vs. 31% LT‐muscular, 21% vs. 17% HT‐muscular, 31% vs. 33% mucosal, 21% vs. 19% muscular‐mucosal, *P* = 0.48). Thus, characterized neurons from both sexes were pooled together and displayed as pie charts in Figure 5. For the following topological study, neurons from both sexes were also pooled together.

**Figure 6:**
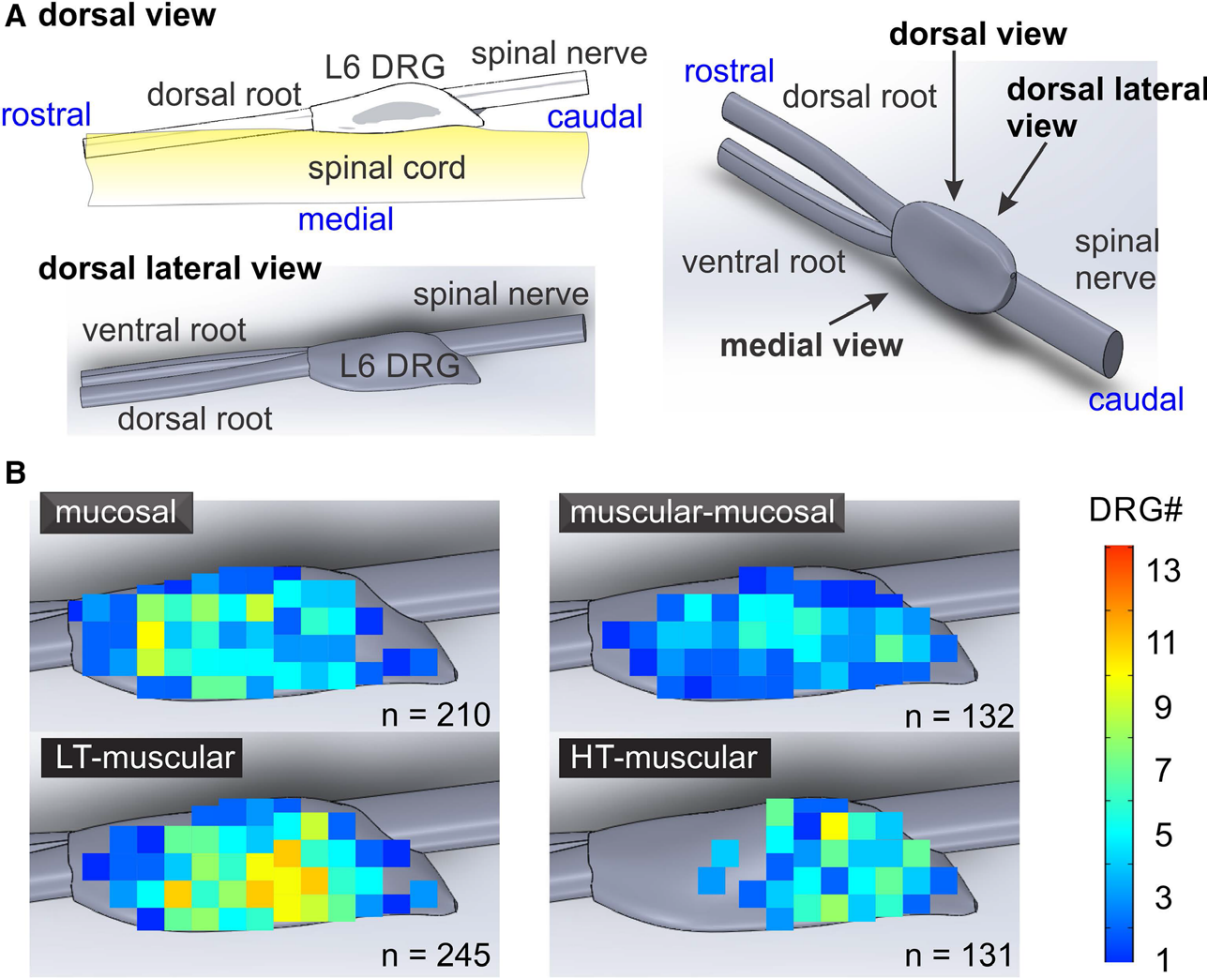
Topological distribution of 718 functionally‐classified colorectal afferents in L6 DRG. (A) The GCaMP6f signals were captured from the dorsal lateral view of L6 DRG. (B) Topological distribution of four colorectal afferent classes in L6 DRG, mucosal, LT‐muscular, muscular‐mucosal and HT‐muscular. LT, low threshold; HT, high threshold.

The large number of characterized L6 DRG neurons (718) permits a topological analysis of colorectal afferents in the L6 DRG. As shown in Figure 6A, GCaMP6f recordings were conducted on the dorsal lateral view of the L6 DRG, which was evenly divided into 60 grids to assess the topological distributions of the four colorectal afferents classes in Figure 6B. Distribution of mucosal, LT‐muscular and muscular‐mucosal afferents are widespread in the L6 DRG. In contrast, HT‐muscular afferents are clustered towards the caudal portion of the L6 DRG (Fig. 6B).

## Discussion

In this study, we implemented a new optical approach to functionally characterize hundreds of colorectal afferent neurons and determined their topological distributions in the lumbosacral DRG in an ex vivo preparation with colorectum, pelvic nerve, and DRG in continuity. Using GCaMP6f, an ultrasensitive and rapid genetically encoded calcium indicator, we show that DRG neural activities can be recorded at a resolution of individual action potentials with a regular upright fluorescent microscope and a high‐speed ultra‐low noise sCMOS camera. The use of a water immersion 10× objective with high numerical aperture ensures that the whole DRG can be captured in the field of view at an ultra‐high frame rate (100 frames/sec), and monitoring neural activities from 600 to 2000 neurons in one DRG simultaneously. The frame rates of recording in previous studies are generally below 14 frames per second. Using similar calcium indicators (e.g., GCaMP3 or GCaMP6s), functional characterization of cutaneous sensory neural activities has been done previously (Emery et al. 2016; Kim et al. 2016; Smith‐Edwards et al. 2016; Chisholm et al. 2018), but the long recovery time (over seconds) of those calcium indicators challenges the interpretation of consecutive action potential spikes from the GCaMP transients. The calcium indicator GCaMP6f with faster recovery time has been used by a recent study to characterize sensory afferents innervating the facial skin, which shows great potential for resolving individual action potentials (Ghitani et al. 2017).

To the best of our knowledge, this is the first study that utilizes GCaMP6f to functionally characterize afferents innervating the viscera. From our prior single‐fiber recording of pelvic nerves (Feng et al. 2010, 2012b,2012c, 2013, 2016; Tanaka et al. 2011; La et al. 2012; Feng and Gebhart 2015), the firing rate of colorectal afferents at threshold input stimuli is much lower than many somatic counterparts, generating only a few action potential spikes to the stimulus of seconds‐long duration. Thus, in order to characterize visceral afferents, recording individual action potentials in somata with high temporal resolution is required. This justifies the use of GCaMP6f which, compared to previous generations of GCaMPs, offers ultra‐sensitivity to individual action potentials and rapid recovery time from fluorescent state. We implemented a high image recording rate (100 frames/sec) in our GCaMP6f recordings, which was sufficient to resolve consecutive action potentials. We used electrical stimulation at the attached dorsal root to activate DRG neurons in a predictable and reproducible manner. The close correlation of GCaMP6f transients with individual action potentials was validated by our concurrent optical and electrophysiological recordings from the same DRG neuron (Fig. 1). Using this approach, we confirm the robustness of the evoked GCaMP6f transients by individual action potentials, usually more than 3% of the baseline fluorescent level for an easy post‐hoc data extraction. In our study, GCaMP6f fluorescence usually returns to the baseline level within 1–2 sec in the DRG somata. At stimulation frequencies above 0.5 Hz, temporal summation of the GCaMP6f transients starts to occur with a gradual increase in the baseline fluorescent level. Nonetheless, individual GCaMP6f transients can be resolved up to 4 Hz stimulation. At frequency beyond 4 Hz, the accumulated fluorescence intensity can still serve as a proxy for the rate of action potential firing. However, across different DRG neurons, the magnitude of calcium transients evoked by the same level of neural activities can vary significantly, presumably reflecting variation in expression levels of GCaMP6f as well as different calcium storage and buffering (Gemes et al. 2010). Thus, the use of the temporal spikes (i.e., the GCaMP transients) rather than the fluorescent GCaMP magnitude in this study provides a more robust functional characterization and classification of colorectal afferents.

This is also the first study to functionally characterize a large number, that is, hundreds of colorectal neurons in intact DRG, which are the minorities in lumbosacral DRG making up less than 10% of total DRG neurons even in the most abundant L6 segment (Christianson et al. 2006b). Prior functional characterization of colorectal afferents relied on blind poking of the DRG with a sharp glass electrode, a low yield approach implemented only by a handful of studies. Malin et al. conducted intracellular recordings from DRG somata in a similar ex vivo preparation with colorectum, pelvic nerve and L6 DRG in continuity using a sharp glass electrode, revealing the low‐threshold and high‐threshold populations of neurons to graded colorectal distension (Malin et al. 2009). Hibberd et al. used a similar ex vivo approach and conducted recordings from a CGRP‐Cre mouse line, documenting that 78% of colorectal neurons are positive for CGRP. Neither studies are able to record more than 100 colorectal DRG neurons (Hibberd et al. 2016), insufficient for a topological distribution study. In contrast, our current optical recording approach allows the neural activities to be recorded from one whole DRG when reproducible mechanical stimuli are applied to the attached colorectum. The use of an objective with relatively low magnification (10X) allows a longer depth of field to capture the fluorescent signals 40–70 *μ*m below the DRG surface. We also moved the focal plane at 50 *μ*m to capture all the DRG in the dorsal lateral view, by which 40–60% of the DRG regions could be monitored concurrently, similar to other optical recordings from lumbar DRG (Chisholm et al. 2018) and trigeminal ganglion (Ghitani et al. 2017). Given that 3.3% VGLUT2‐positive neurons innervate the colorectum from our fast‐blue tracing study, the fact that 20–80 colorectal neurons are recorded in each DRG strongly indicates that the GCaMP6f recording can monitor the responses of 600–2400 neurons in the DRG. Also, our GCaMP6f approach in detecting individual action potentials is also robust and reliable with more than 3% change of the baseline fluorescent level following each action potential, in contrast to less than 1% fluorescent changes in optical recordings using voltage‐sensitive dyes (Chemla and Chavane 2010). Collectively by using the GCaMP6f and a 10× objective, we managed to characterize 791 lumbar colorectal neurons in 14 VGLUT2/GCaMP6f mice, a dramatic improvement in efficiency compared to the previous approach with sharp electrode recordings.

In this study, the colorectum was kept in the tubular shape and subjected to two distinct mechanical stimuli for functional afferent characterization: graded luminal distension from non‐noxious (<25 mmHg) to noxious range (>25 mmHg) and luminal shear flow (20–30 mL/min). This is different from the prior approach by us and others with colorectum cut open along the mesentery and pinned flat for easy access to afferent receptive fields (Brierley et al. 2004; Feng and Gebhart 2011; Feng et al. 2015, 2016; Zhu et al. 2015). Single‐fiber recordings from teased pelvic nerve filaments are less affected by the slight movement and perturbation of nerve fibers from the manual manipulations of the receptive fields in the flattened colorectum, for example, von Frey probing, mucosal stroking, and circumferential stretch. However, optical recordings of GCaMP6f transients require the translational movement of the DRG to be no more than a few microns, a fraction of the DRG neural diameter. To achieve that, we removed the human manipulation by developing computer‐controlled valve systems for applying the graded colorectal distension and luminal shear stimuli, which was qualitatively equivalent to the circumferential stretch and mucosal stroking in the previous stimuli (Feng and Gebhart 2015). The proportion of muscular, mucosal and muscular‐mucosal afferents identified in the current study generally agrees with the previous studies of flattened colorectum (Feng and Gebhart 2011; Feng et al. 2012b,2012c; La et al. 2012), except that the mucosal and muscular‐mucosal proportions are slightly smaller, likely reflecting the weaker stimulus of luminal shear flow than the 10 mg mucosal stroking. The serosal afferents activated by punctate probing of the receptive field beyond the physiological stress range (Feng et al. 2010) are likely not characterized by the current stimuli. However, the term “serosal” appears to be a misnomer(Feng et al. 2012a) as a recent study shows that almost no nerve endings are located in the colorectal serosa (Spencer et al. 2014). Keeping the colorectum in tubular shape prevents the characterization of mechanically‐insensitive afferents (MIAs), which were previously identified and characterized by us with focal electrical stimulation to the receptive fields in the flattened colorectum (Feng and Gebhart 2011; Feng et al. 2016). A recent study indicates those MIAs appear to express nicotinic acetylcholine receptor subunit alpha 3 (Chrna3) (Prato et al. 2017). Future studies could focus on the MIAs by selective expression of GCaMP6f in those Chrna3‐positive DRG neurons. The high‐threshold (HT) muscular afferents that encode noxious colorectal distension (>25 mmHg) are putative nociceptors to evoke visceral pain (Feng et al. 2016). In the current study, the proportion of HT‐muscular afferents in all stretch‐sensitive afferents (25.8%) is in agreement with the range of 14–25% as reported by prior studies via single‐fiber recordings (Sengupta and Gebhart 1995; Feng et al. 2016). In summary, the current approach of using graded colorectal distension of luminal shearing allows us to effectively characterize a putative nociceptive (HT‐muscular) and three other afferent classes in the lumbosacral DRG.

This is also the first study to reveal the topological distribution of the four classes of DRG neurons in the L6 DRG. It is striking to see that the putative colorectal nociceptors, that is, HT‐muscular afferents are clustered toward the caudal portion of the DRG whereas the other three afferent classes that encode non‐noxious stimuli spread out throughout the L6 DRG. We recognize two limitations of the current study in regards to cell distribution. First, approximately 600–2400 neurons close to the dorsal lateral surface were monitored for responses to colorectal distention in each DRG, which comprise less than 25% of the total neuron numbers in mouse lumbosacral DRG. Thus, the topological distribution will likely reflect colorectal neurons in superficial layers, which are likely the target of non‐invasive DRG neuromodulation with surface electrodes situated outside the dura mater. Second, only VGLUT2‐expressing neurons are characterized in this study, which makes up 97% of lumbosacral colorectal afferents from a prior immunostaining study (Brumovsky et al. 2011) and more than 78% from our current study (Fig. 1). It is unclear regarding the role of the small proportion of VGLUT3‐ or VGLUT1‐expressing colorectal neurons in visceral hyperalgesia which awaits further investigations. A study on the cutaneous mechanical pain suggests that VGLUT3‐expressing DRG neurons do not contribute to behavior mechanical hyperalgesia whereas VGLUT3‐expressing spinal dorsal horn neurons do (Peirs et al. 2015).

Our findings of clustering of putative colorectal nociceptors in the caudal region of the DRG could have significant implications for the design of DRG neuromodulations that target lumbosacral DRG for managing IBS‐related visceral pain. There are several obvious advantages to targeting the DRG. First, the confined location of DRG in inter‐vertebral foramina can significantly reduce electrode lead movement due to changes in patient posture (Zhang et al. 2014). Second, DRG are located outside the enclosure of cerebrospinal fluid, and are less likely to be influenced by patient posture. Third, DRG contain the somata of sensory afferent neurons only, thus permitting selective neuromodulation of the sensory innervation by avoiding the motor efferents. Fourth, afferent sensitization correlates with the pathophysiology of the somata in the DRG, including changes in excitability (Gold and Flake 2005), gene expression (Zhang et al. 1998; Obata and Noguchi 2004, 2006; Miller et al. 2009), membrane and cytosol protein levels (Gold 1999), and glial cell functions (Schaeffer et al. 2010). Thus, DRG neuromodulation could have a profound therapeutic impact that outlasts the duration of stimulation (Pope et al. 2013). Thus, outcomes from this study and future studies using optical electrophysiology to characterize colorectal DRG in health and disease will have great impacts on the non‐drug management of IBS‐related visceral pain via DRG neuromodulation. In particular, the function and topology of colorectal DRG neurons in the context of prolonged visceral pain await further investigations in long‐term mouse models of IBS.

In summary, we utilized an optical electrophysiology approach to conduct high‐throughput recordings from mouse colorectal afferents in the lumbosacral DRG. Using a high‐speed ultra‐low noise sCMOS camera and self‐developed plug‐in program to automatically analyze evoked GCaMP6f transients, we characterized the functional responses of 791 colorectal neurons to graded colorectal distension and luminal shear flow. We also revealed for the first time that high‐threshold muscular afferents are clustered in the caudal region of the L6 DRG. Further research using this established optical recording approach is required to reveal changes of topological distributions of DRG neurons in the context of peripheral afferent sensitization in prolonged visceral pain.

## Conflict of Interest

The authors claim no conflict of interests.
